# Efficacy of PEERS® for Adolescents via Telehealth Delivery

**DOI:** 10.1007/s10803-022-05580-5

**Published:** 2022-05-27

**Authors:** Jasper A. Estabillo, Christine T. Moody, Solene J. Poulhazan, Laura H. Adery, Elizabeth M. Denluck, Elizabeth A. Laugeson

**Affiliations:** 1 Department of Psychiatry and Biobehavioral Sciences, Semel Institute for Neuroscience and Human Behavior, University of California, 300 UCLA Medical Plaza, Los Angeles, CA 90095-6967 USA; 2 Department of Psychology, University of California, Los Angeles, CA USA; 3 College of Education School Psychology Program, University of Oregon, Eugene, OR USA

**Keywords:** PEERS®, Social skills intervention, Autism spectrum disorder, Telehealth

## Abstract

PEERS® for Adolescents is an evidence-based social skills intervention for autistic youth and adolescents with other social challenges. The efficacy and effectiveness of PEERS® are well established; however, limited data on PEERS® via telehealth delivery exist. The current study aimed to examine the efficacy of PEERS® for Adolescents via telehealth and compare outcomes between telehealth and in-person modalities. Thirty-one adolescents (*M*_*age*_ = 13.77, *SD* = 2.14) participated in telehealth groups, and outcomes were compared with 212 adolescents (*M*_*age*_ = 14.02, *SD* = 2.00) from in-person groups. Findings demonstrate PEERS® for Adolescents via telehealth results in significant improvements in social skills knowledge, social responsiveness, overall social skills and problem behaviors, and social engagement. Telehealth outcomes are relatively equivalent to in-person delivery.

The UCLA PEERS® for Adolescents program is an evidence-based, parent-assisted social skills group therapy intervention originally developed for autistic^1^ youth (Laugeson et al., 2009). The program utilizes evidence-based methods for teaching social skills to autistic adolescents, including small group format, didactic instruction, role play models, behavioral rehearsal, and generalization homework assignments (Moody & Laugeson, 2020). Extensive research and meta-analytic findings support the use of PEERS® for Adolescents for youth on the autism spectrum (Zheng et al., 2021) and with other neurodevelopmental disorders (Gardner et al., 2019; Wolstencroft et al., 2021; Wyman & Claro, 2020). Previous studies have demonstrated that after completing the PEERS® for Adolescents program, adolescents show improved social skills and social engagement, as well as reduced problem behaviors, autism spectrum disorder (ASD)—related social difficulties, and social anxiety (Laugeson et al., 2012; Schohl et al., 2014). These gains maintain well after the treatment has ended (Mandelberg et al., 2014), a finding attributed to parental involvement in the intervention (i.e., simultaneous acquisition of PEERS® skills content and training in effective social coaching).

Given the salience of peer interactions and affiliations during the adolescent developmental period, evidence-based interventions that support youth social functioning are essential. In the broader population, adolescence is a time of shifts away from family systems and toward peers, and youth strive for independence and individual identity formation (Steinberg & Morris, 2001). Social struggles and peer victimization in childhood and adolescence are predictive of later mental health problems, emotional dysregulation, academic difficulties, poor physical health, and delinquency (Bierman et al., 2015; Fussner et al., 2018; Moore et al., 2017). Autistic youth have been found to be more likely to experience peer victimization and loneliness than their neurotypical peers and peers with other disabilities (Deckers et al., 2017; Forrest et al., 2020; Park et al., 2020), possibly due to differences in social communication and attention-narrowing behaviors characteristic of autism (APA, 2013). In addition to the broad social adjustments of adolescence, these particular social challenges confer additional risk for negative outcomes in an already vulnerable population (Rodriguez et al., 2021). Social skills training allows socially motivated adolescents on the autism spectrum to mitigate such social challenges and corresponding adverse outcomes by offering additional support and guidance in decoding their social landscape.

Beyond the typical developmental processes, the most recent generational cohort of adolescents has faced unique challenges in adapting to and interfacing with the rising integration of electronic communication into society over the past two decades. Research indicates that online interaction (e.g., text messages, social media, video chats) among adolescents has increased, while time spent engaging in in-person interactions has significantly declined (Twenge et al., 2019). Although autistic youth spend significantly more time than their allistic peers “on screens” (e.g., playing computer games or watching videos) (Slobidin et al., 2019) data suggest they spend less time engaging in computer-mediated communication than their peers (Paulus et al., 2019). Despite this difference, autistic adults were significantly more likely than adults without ASD to endorse social benefits of electronic communication, such as increased time to think, practice social interactions, and ability to express one’s true self (Gillespie-Lynch et al., 2014).

Overall, online communication and social networking in adolescence appears to produce both potential benefits and risks for neurotypical adolescents (Best et al., 2014) and autistic youth (Macoun et al., 2021). For example, data suggest that online communication with friends can reduce emotional distress and increase feelings of closeness among friends (Dolev-Cohen & Barak, 2013; Valkenburg & Peter, 2007a). For adolescents on the spectrum, social media use and electronic communication have been correlated with more friendships and better friendship quality (Kuo et al., 2014; van Schalwyk et al., 2017). With respect to risks, adolescent communication with strangers on the internet has adverse effects on well-being, especially for adolescents who endorse high levels of loneliness (Valkenburg & Peter, 2007b). Adolescent social media use has also been related to increased engagement in risky behaviors, including substance use and sexual activity (Vannucci et al., 2020). Additionally, electronic communication has produced a novel context for peer victimization to occur via cyberbullying (Kowalski et al., 2012). Although estimates vary, some studies indicate that more than half of adolescents report having been a victim of cyberbullying in the past year (Brochado et al., 2017). For autistic youth, one study found 12.5% of autistic adolescents reported being the victim of cyberbullying (Kloosterman et al., 2013).

Technology platforms have also been increasingly used for positive mean, such as expanding service delivery within the medical and mental health fields. One advantage of remote service delivery via telehealth is the opportunity to address disparities in access to services, particularly inequalities across geographic locations and socioeconomic levels. Such disparities are prominent in autism-related services, with fewer service providers specializing in ASD available in rural and low-income areas (Drahota et al., 2020). Initial research on telehealth services for autism included parent-mediated interventions and applied behavior analysis (ABA) approaches for young children, and studies showed benefits following telehealth delivery of these treatments (Ferguson et al., 2019; Sutherland et al., 2018; Vismara et al., 2013). However, fewer designs include direct comparisons to in-person modalities which limits interpretation of outcomes. One such study found that ABA interventions that target challenging behaviors in young children with ASD via telehealth produce similar gains to in-person treatment and with lower financial costs (Lindgren et al., 2016).

The initial research on interventions for young children with ASD is promising; however, very few telehealth services for adolescents on the spectrum and their families have been systematically investigated. A pilot study of a group cognitive behavioral intervention for anxiety with autistic adolescents found positive improvements to youth anxiety symptoms and parents’ sense of competence, as well as high family satisfaction with the telehealth modality (Hepburn et al., 2016). Another study utilized a private Facebook group as a supplementary follow-up service to an in-person adolescent social skills training group, aiming to reinforce and generalize social skills to a social media context. Despite all six participants in the study reporting satisfaction and perceived opportunities to practice skills in the Facebook group, no improvements on standardized measures of social skill mastery were observed (Gwynette et al., 2017). Although not peer-reviewed, a small pilot study of a telehealth translation of PEERS® for Adolescents showed improved social functioning and decreased problem behaviors for four of the five participants (Miyake et al., 2018).

As technology becomes increasingly intertwined with all facets of life and social interaction for much of the world, clearly a more robust effort to explore the potential for telehealth as a treatment modality for autistic youth is necessary. In particular, given the increase in social communication and activity occurring online, social skills training groups may have particular advantages when provided via a telehealth format. Teaching and practicing social skills via telehealth and assigning in-person homework assignments may support generalization across multiple contexts, a process that is traditionally challenging for individuals on the autism spectrum (Barry et al., 2003). However, beyond simply transferring current social skills programs to an online format, it is also essential for social skills curricula to incorporate adapted and novel skills unique to the increasingly virtual social landscape. Though much of the currently available research has focused on social media and networking, an exponential rise in the use of videoconferencing technology has also resulted in a new social milieu, requiring adaptations to social communication behaviors. For example, researchers have highlighted differences in norms and availability of nonverbal communication information while videoconferencing (e.g., extended experience of eye gaze, lack of body language cues) and have begun theorizing the impacts of these differences on our social cognitive functioning (Bailenson, 2021; Wiederhold, 2020). Decoding the rules and nuances of online communication in social skills programs is especially important given findings that individuals who are less socially skillful tend to prefer to communicate online rather than in-person (Kang & Munoz, 2014).

In sum, despite some preliminary positive findings, investigations of telehealth services for youth on the spectrum have been sparse and yet to determine efficacy. Given that literature in this area is in its infancy, the availability and dissemination of such services in clinical settings has been minimal. However, prompted by the health risks posed by the novel coronavirus in early 2020 and further supported by emergency shifts in insurance reimbursement policies in the United States, a rapid transition from in-person clinical service provision to remote delivery has led to an increase in the implementation and opportunity for examination of telehealth services.

In response to the COVID-19 pandemic, the UCLA PEERS® Clinic transitioned clinical services to telehealth implementation, utilizing HIPAA-compliant Zoom videoconferencing, PowerPoint slides for didactic content, pre-recorded role play videos, and breakout room technology to replicate the structure and format of our programs. Additionally, given the pervasive use of online communication in adolescents’ daily lives, which was further accelerated by remote schooling and social distancing policies, additional didactic elements related to online social etiquette were incorporated into the program to supplement existing content. The current study aimed to: (1) test the efficacy of the telehealth adaptation of the UCLA PEERS® for Adolescents program by examining changes in social functioning over the course of the 16 week group-based treatment and (2) compare efficacy between PEERS® for Adolescents via telehealth delivery to the original, in-person program to determine potential differences in treatment response based on service modality.

## Method

### Participants

This study was approved by the UCLA Institutional Review Board, and all procedures were performed in accordance with the approved IRB. The participants included adolescents and their parents who enrolled in the UCLA Clinic’s PEERS® for Adolescents Program between June 2015 and June 2021. Eligibility requirements for enrollment in the clinical groups included: (a) enrollment in middle or high school, (b) adolescent motivation and willingness to participate in treatment, (c) presence of significant social challenges, (d) absence of significant other mental health treatment priorities (e.g., not currently or recently hospitalized for suicidal ideation), (e) capacity to meaningfully participate in and understand group lessons, as determined by clinical judgment (including level of cognitive functioning and presence of significantly interfering challenging behaviors), (f) fluency in English, and (e) presence of a parent or caregiver also fluent in English and willing to participate in the group as the adolescent’s social coach. Data obtained from intervention groups were entered into an archival database that continues to expand with ongoing clinical services.

For the purposes of the current study, only data from adolescents with historical diagnoses of ASD and/or clinically elevated autism symptoms on the Social Responsiveness Scale, Second Edition (Constantino & Gruber, 2012) were included in the analyses. The telehealth group included 31 youth (*M*_*age*_ = 13.77, *SD* = 2.14, 64.5% male) who participated in the program between May 2020 and June 2021. The 31 adolescents in the telehealth group were selected from those who met study criteria from a larger sample of 93 youth who enrolled in the telehealth program (including those without ASD), of which 71 youth completed the telehealth intervention and 22 youth dropped. Approximately 2–3 adolescents dropped per 12 person cohort (*M* = 2.75), which is comparable to the number of drops per in-person cohort. Reasons for dropping from the telehealth program were similar to in-person reasons (e.g., adolescent and/or family scheduling conflicts). Of the 71 telehealth completers, only 36 youth completed parent and/or adolescent baseline and post-intervention measures, and five adolescents were excluded from the current study due to not having an ASD diagnosis and/or a clinically elevated SRS-2. The comparison in-person group included 212 adolescents (*M*_*age*_ = 14.02, *SD* = 2.00, 72.6% male) who participated in the program between June 2015 and October 2019. The 212 adolescents in the in-person group were from a total of 297 youth who enrolled in the in-person program, of which 220 youth completed the program and 77 youth dropped. Of the full in-person cohort, data from 242 adolescents have been scored, verified, and entered into our database to be available for analyses. An additional 30 participants were excluded from analyses due to not having an ASD diagnosis and/or clinically elevated SRS-2. Demographic information is shown in Table 1.

**Table 1:** Demographic characteristics for telehealth and in-person groups

Variable	Telehealth (*n* = 31)			In-person (*n* = 212)		
	*M*	*SD*	%	*M*	*SD*	%
Age	13.7	2.1		14.0	2.0	
Gender						
Female			35.4			26.8
Male			64.5			72.6
Ethnicity						
White			58.0			58.9
Latinx or Hispanic			6.4			8.0
African American or Black			3.2			1.4
Asian			16.1			8.0
Native American			0			0.4
Middle Eastern			0			2.8
Multiracial			12.9			15.0
Other			3.2			3.3

## Procedures

All participants, accompanied by their parents, completed an intake eligibility appointment with a postdoctoral fellow or licensed clinical psychologist prior to the first session. Participants completed all outcome measures (described below) at baseline and at the conclusion of the intervention. For in-person groups, all participants consented and completed hard copy protocols at time of intake for baseline and at the last session for post-intervention measures. For telehealth groups, measures were administered through a secure online survey platform [UCLA Qualtrics] in which adolescent and parent participants completed a battery of measures on the youth’s social functioning. Baseline measures were collected prior to the first treatment session, and post-intervention measures were completed after session 15 and up to 3 weeks post-intervention.

### Intervention

PEERS® for Adolescents is a manualized, parent-assisted social skills intervention for autistic youth and adolescents with other social challenges that teaches skills related to making and keeping friends and handling peer rejection and conflict (Laugeson, 2014; Laugeson & Frankel, 2010). The PEERS® for Adolescents program consists of 16 weekly 90 min sessions focused on different topics and skills each week. During the in-person delivery, youth and their parents attended separate, concurrent sessions in an outpatient clinic that instructed them on key elements about friendships. Adolescent and parent groups were led by clinicians (e.g., licensed clinical psychologists, clinical psychology postdoctoral fellows, clinical psychology pre-doctoral interns) with previous experience conducting social skills groups for youth and expertise in working with individuals on the autism spectrum. Trained and supervised behavioral coaches (e.g., clinical psychology pre-doctoral interns, graduate students, post-baccalaureate and undergraduate research assistants) assisted with role play demonstrations, behavioral rehearsals, providing performance feedback through coaching, monitoring homework compliance, and maintaining treatment fidelity. For telehealth delivery, all groups were conducted with the same procedures (i.e., 16 week PEERS® for Adolescents manualized program, 90 min sessions, separate concurrent adolescent and parent groups, same level of training and expertise of group leaders, similar types and ratio of clinical staff supporting participants) with the exception of all group members, clinicians, and behavioral coaches utilizing HIPAA-compliant Zoom Video Communications to participate rather than meeting together face-to-face in conference rooms. Written consent for telehealth services was obtained prior to the initiation of the program, and verbal consent was given by each participant at the start of each session.

PEERS® didactic lessons were taught to the group through instruction of concrete rules and steps for ecologically valid social skills based on norms established by socially successful youth. Lessons include: (a) conversational skills (two sessions), (b) electronic communication, (c) sources of friends, (d) appropriate use of humor, (e) starting and entering conversations, (f) exiting conversations, (g) good sportsmanship, (h) get-togethers, (i) handling disagreements, (j) changing bad reputations, (k) handling teasing and embarrassing feedback, (l) avoiding physical bullying, (m) handling cyberbullying, (n) handling rumors and gossip, and (o) graduation and moving forward. Telehealth groups also included newly developed content covering concordant skills relevant to online social interactions during regular session instruction [e.g., starting and ending video chats (lesson e), entering and exiting conversations online (lesson f), having online get-togethers (lesson h)]. During in-person sessions, group leaders and coaches conducted live role play demonstrations of appropriate and inappropriate social behaviors in order to use Socratic questioning with youth to generate ecologically valid rules and steps for more appropriate social interactions and to practice perspective taking to enhance social cognition. Adolescents then practiced newly learned skills through structured socialization activities during which they received in vivo coaching from the treatment team. To promote generalization of skills, adolescents were assigned weekly socialization homework assignments to practice skills with their parents and peers. Parents were instructed on social coaching techniques to promote skills mastery and to assist their adolescent with social problem solving. For telehealth groups, didactic content was presented utilizing PowerPoint slides, role plays were provided by pre-recorded videos, and behavioral rehearsals were facilitated by coaches in small group (3–4 youth) virtual breakout sessions. In addition to the regular weekly assignments given to the in-person groups, telehealth groups were also assigned in-group (i.e., with other program participants) online get-togethers during sessions 9–15 of the program. This change was made for the purpose of practicing online social skills and reducing ongoing negative impacts of social isolation due to COVID-19, particularly during remote schooling.

## Measures

### Test of Adolescent Social Skills Knowledge (TASSK; Laugeson & Frankel, 2010)

The TASSK is a 30-item criterion-referenced measure developed to assess changes in knowledge about social skills taught in the PEERS® intervention. The TASSK takes approximately 5 minutes for youth to complete. Scores are calculated out of a total of 30, where higher scores reflect greater knowledge of adolescent social skills. The TASSK has shown to be sensitive to treatment effects and has a moderate coefficient alpha of 0.56, which is acceptable given the large domain of questions included on the scale (Laugeson et al., 2009). Adolescents completed the TASSK at baseline and post-intervention.

### Social Responsiveness Scale, Second Edition-School Age (SRS-2; Constantino & Gruber, 2012)

The SRS-2 is a 65-item measure of the presence and severity of ASD-associated social challenges and symptomatology in individuals 2.5 years old through adulthood. The SRS-2 offers four forms [e.g., School-Age, Preschool, Adult (Relative/Other Report), and Adult (Self-Report)], and the current study utilized the School-Age form, which is completed by caregivers to report on individuals four to 18 years of age. The SRS-2 takes approximately 15–20 min to complete. Parents rated their adolescent’s behaviors using a 4-point Likert style scale ranging from 1 (“*not true*”) to 4 (“*almost always true*”). The measure produces a standardized Total T-score and five subscale scores [e.g., Social Communication, Social Cognition, Social Awareness, Social Motivation, and Restricted Interests and Repetitive Behavior (RRB)], with higher scores reflecting greater socialization difficulties. T-scores above 60 are categorized in the clinical level, with scores 60 to 65 in the Mild range, 66 to 75 in the Moderate range, and 76 and higher in the Severe range. Scores 59 and below are within normal limits and not typically associated with ASD. Psychometric properties of the SRS-2 show excellent internal consistency (ranging from 0.94 to 0.96 across age groups), interrater reliability correlations of 0.77 for the School-Age form, and moderate to high correlations with other measures of social behavior and communication and inter-rater agreement (coefficients ranging from 0.72 to 0.82) (Bruni, 2014). The SRS-2 was completed by caregivers at baseline and post-intervention.

### Social Skills Improvement System Rating Scales (SSiS; Gresham & Elliott, 2008)

The SSiS is a 79-item measure that assesses general social skills and interfering problem behaviors in children three to 18 years of age. Parents reports on their adolescent’s behaviors on a 4-point scale ranging from 0 (“*never*”) to 3 (“*almost always true*”). Standard scores are available for the overall domains of Social Skills and Problem Behaviors, with higher scores indicating better social functioning and greater difficulties with behavioral problems, respectively. Standard scores are classified as the following: 116 and greater = Above Average, 85–115 = Average, 70–84 = Below Average, and 69 and below = Well Below Average (Gresham & Elliot, 2008). Parent forms have high internal reliability with alpha coefficients in the mid to high 0.80 s for overall domain standard scores. Caregivers completed the SSiS at baseline and post-intervention.

### Quality of Socialization Questionnaire (QSQ; Laugeson & Frankel, 2010)

The QSQ is a 12-item measure that assesses the frequency of an individual’s social engagement (i.e., get-togethers with peers) and level of conflict during those get-togethers (Laugeson et al., 2009, 2012). The QSQ is adapted from the Quality of Play Questionnaire (QPQ; Frankel & Mintz, 2011) for adolescents and young adults on the spectrum and takes approximately 2–3 min to complete. The QSQ measures an individual’s frequency of get-togethers (i.e., hosted and invited) in the previous month. The QSQ was completed by adolescents (self-report) and caregivers (parent-report) at baseline and post-intervention.

## Statistical Analyses

All statistical analyses were conducted with SPSS 27.0. A priori analyses explored potential group differences with respect to demographic variables. Independent samples t-tests indicated no significant differences between telehealth and in-person groups on age, *t*(240) = 0.63, *p* > 0.05. Chi-square analyses also revealed no significant differences between groups on gender, χ^2^(2) = 1.11, *p* > 0.05, or race/ethnicity, χ^2^(7) = 3.64, *p* > 0.05. A priori analyses were also conducted to examine potential group differences on baseline scores on each outcome measure. No significant differences were found between telehealth and in-person groups on baseline scores on the TASSK, SRS Total T-score, SSiS Social Skills Standard Score, SSiS Problem Behaviors standard score, QSQ self-report (i.e., total, hosted, and invited), or QSQ parent-report (i.e., total, hosted, and invited), with all *p* values > 0.05.

To examine the efficacy of telehealth delivery on measures of social functioning, a series of repeated measures t-tests were conducted to compare total scores on each measure from baseline to post-intervention. To compare efficacy between telehealth and in-person groups, difference scores (DS) were calculated for each participant on outcome measures to show the magnitude of change from baseline to post-intervention. Positive DS on the TASSK, SSiS Social Skills standard score, and QSQ indicated improvements on their respective measures, while negative DS on the SRS Total T-score and SSiS Problem Behaviors standard score indicated improvements on their respective measures. A series of independent samples t-tests were then conducted to compare the telehealth and in-person groups, with DS from baseline to post-intervention on each respective measure as the dependent variable.

## Results

**Fig. 1:**
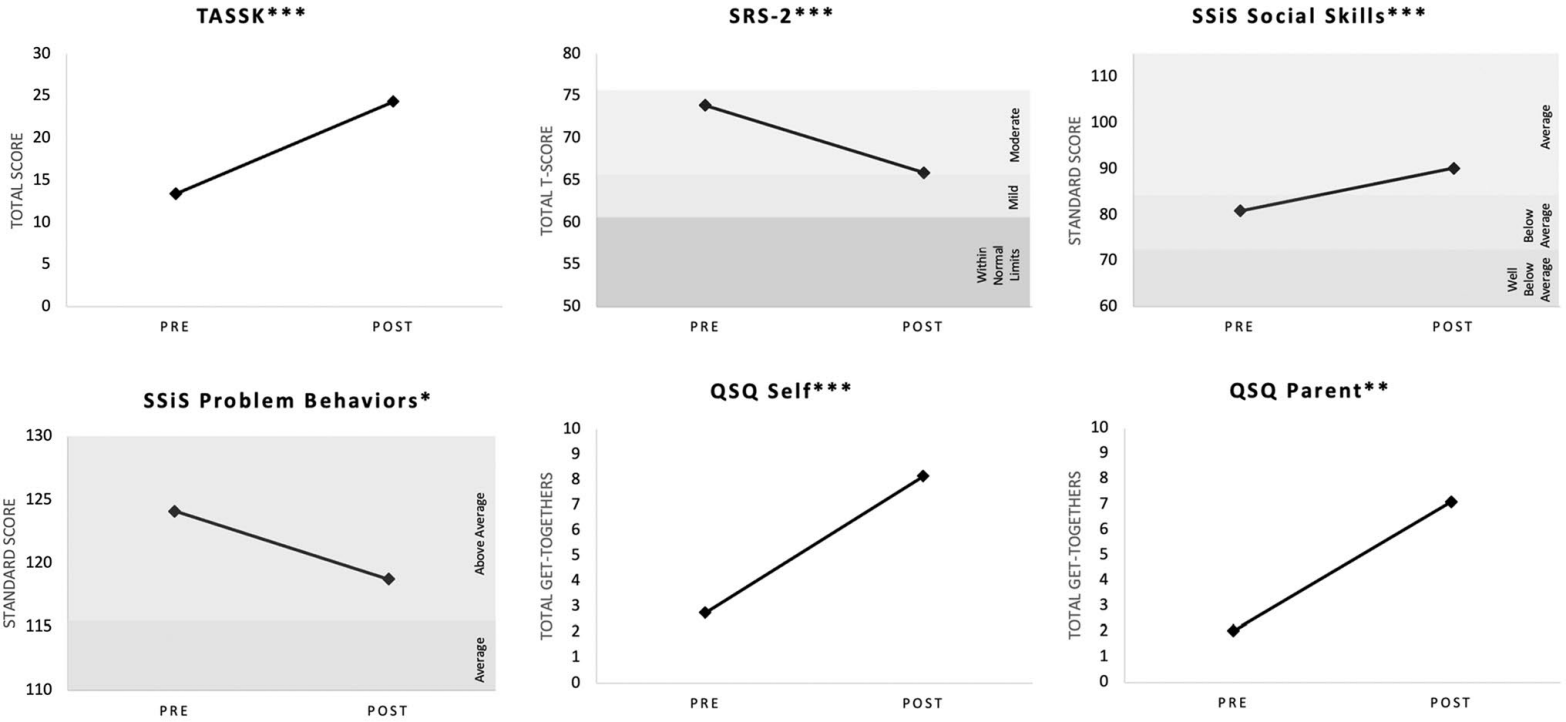
Change in treatment outcome scores from baseline to post-intervention for telehealth group *p < 05, **p < 01, ***p < 001

Overall results indicated significant improvements on all youth outcomes for PEERS® for Adolescents via telehealth delivery from baseline to post-intervention. Results are shown in Fig. 1. Comparison of telehealth and in-person DS showed no significant differences in treatment response, with the exception of parent report of social engagement. See Table 2.

**Table 2:** Summary of treatment outcomes for telehealth and in-person groups

Measure	Telehealth (*n* = 31)		In-person (*n* = 212)		Difference scores t-test
	Difference scores		Difference scores		*P* value
	*M*	*SD*	*M*	*SD*	
TASSK	10.90	5.00	10.58	5.90	n.s.
SRS Total T-score	− 8.00	8.76	− 7.87	9.29	n.s.
SSiS Social Skills Standard Score	9.21	10.50	8.51	10.80	n.s.
SSiS Problem Behaviors Standard Score	− 5.34	11.01	− 8.71	11.46	n.s.
QSQ Self Total	5.40	5.02	3.23	6.12	n.s.
QSQ Parent Total	5.07	7.94	2.47	3.70	< .01

*n.s.* not significant

### Telehealth Efficacy

#### Social Skills Knowledge

Adolescents in the telehealth group were found to significantly improve their social skills knowledge from baseline to post-intervention, *t*(19) = − 9.74, *p* < 0.001. At baseline, youth were responding to about half of the TASSK correctly (*M* = 13.45, *SD* = 3.15), and at post-intervention, scores increased by an average of 11 points (*M* = 24.35, *SD* = 3.90).

#### Social Responsiveness

Adolescent social responsiveness on the SRS-2 Total T-scores significantly improved following telehealth treatment, *t*(28) = 4.92, *p* < 0.001. At baseline, youths’ Total T-score was in the Moderate range (*M* = 73.93, *SD* = 10.89), and at post-intervention, scores decreased to the Mild range (*M* = 65.93, *SD* = 8.51).

#### Overall Social Skills and Problem Behaviors

Adolescent overall social skills, as measured by the SSiS Social Skills standard score, also significantly improved from baseline to post-telehealth intervention, *t*(28) = − 4.73, *p* < 0.001. At baseline, scores were in the Below Average range (*M* = 80.97, *SD* = 11.22) and improved to the Average range at post-intervention (*M* = 90.17, *SD* = 12.18). Further, adolescents’ SSiS Problem Behaviors standard scores significantly decreased from baseline (*M* = 124.10, *SD* = 14.79) to post-intervention (*M* = 118.76, *SD* = 13.65), *t*(28) = 2.61, *p* < 0.05; however, scores still remained in the Above Average range following PEERS® for Adolescents via telehealth.

#### Social Engagement

Adolescent report of total number of get-togethers in the previous month significantly increased from baseline (*M* = 2.75, *SD* = 2.90) to post-intervention (*M* = 8.15, *SD* = 5.19), *t*(19) = − 4.81, *p* < 0.001. Further analyses with Bonferroni corrections to adjust for multiple comparisons revealed adolescents to significantly increase both number of hosted (baseline: *M* = 1.65, *SD* = 2.23; post-intervention: *M* = 5.65, *SD* = 4.06; *t*(19) = − 4.31, *p* < 0.001) and invited get-togethers (baseline: *M* = 1.1, *SD* = 1.21; post-intervention: *M* = 2.50, *SD* = 2.35; *t*(19) = − 2.57, *p* < 0.025). Parent report of total number of get-togethers in the previous month also significantly increased from baseline (*M* = 2.03, *SD* = 5.57) to post-intervention (*M* = 7.10, *SD* = 5.51), *t*(28) = − 3.44, *p* < 0.01. Further examination with Bonferroni corrections to adjust for multiple comparisons revealed parents reported significantly increased number of hosted get-togethers (baseline: *M* = 1.00, *SD* = 2.85; post-intervention: *M* = 4.59, *SD* = 3.04; *t*(28) = − 4.46, *p* < 0.001) but not invited get-togethers (baseline: *M* = 1.03, *SD* = 2.82; post-intervention: *M* = 2.52, *SD* = 2.79, *t*(28) = − 2.07, *p* > 0.025).

### Telehealth and In-person Comparison

The in-person comparison group showed significant improvements on all outcome measures from baseline to post-intervention, all *p* values < 0.05. When comparing telehealth and in-person groups, no significant differences in DS on social skills knowledge, social responsiveness, general social skills, problem behaviors, or teen-report of social engagement were found across treatment modalities, all *p* values > 0.05. DS on parent-report of number of total get-togethers, however, was significantly different between telehealth (*M* = 5.07, *SD* = 7.94) and in-person groups (*M* = 2.47, *SD* = 3.70), *t*(155) = − 2.60, *p* < 0.01. Further analyses of parent-report of get-togethers at post-intervention indicated significant differences between groups on both number of hosted (telehealth: *M* = 4.59, *SD* = 3.04; in-person: *M* = 3.06, *SD* = 2.59; *t*(165) = − 2.80, *p* < 0.01) and invited get-togethers (telehealth: *M* = 2.52, *SD* = 2.79; in-person: *M* = 1.31, *SD* = 1.59; *t*(165) = − 3.20, *p* < 0.01). Significant differences between telehealth and in-person modalities on parent-reported get-togethers consistently favored the telehealth format. Though promising, potential confounds and limitations of the latter finding are discussed below.

## Discussion

Research on telehealth delivery of mental health interventions has increased due to the availability of telehealth platforms and the service context of the COVID-19 pandemic. For autistic individuals, research on telehealth interventions is in its infancy; despite some positive evidence for remote service delivery, there remains a high need for continued research in this domain. Findings from the present study indicate that PEERS® for Adolescents via telehealth delivery is a very promising method to teach autistic youth social skills and improve outcomes. Consistent with previous research (Miyake et al., 2018), the current study found that telehealth delivery results in significant improvements on measures of social functioning. Importantly, the current study found telehealth delivery to not only be efficacious in improving social outcomes, but results were comparable to outcomes via in-person instruction. This finding is especially important given the current context of remote learning and social distancing and provides support for the use of telehealth delivery beyond the COVID-19 pandemic. Therefore, continued use of telehealth services and research on improving the efficacy and effectiveness of remote service delivery will remain to be of importance.

Similar to the in-person program and original studies (Laugeson et al., 2009, 2012), findings showed PEERS® for Adolescents via telehealth delivery improves friendship skills of autistic youth. Results support the use of telehealth instruction to teach and practice social skills, as adolescents showed improvements on measures of social skills knowledge, social responsiveness, general social skills, problem behaviors, and social engagement. As in the in-person group, adolescents who participated in the telehealth format were found to significantly improve social skills knowledge by about 10–11 points. Thus, both telehealth and in-person instruction are effective methods to teach social skills content. Regarding social responsiveness and overall social skills, both telehealth and in-person groups were in the Mild range on the SRS-2 and Average range for SSiS Social Skills at post-intervention. Although significant improvements were found from baseline to post-intervention on SSiS Problem Behaviors, standard scores remained in the Above Average range. This finding may be attributed to the social skills focus of the program rather than targeting specific problem behaviors. Social engagement was also found to significantly improve, and results show that learning and practicing skills on how to have get-togethers, particularly learning the rules and steps for hosting get-togethers contributes to improved social engagement. For the telehealth group, at post-intervention, youth were hosting twice the total number of get-togethers they participated in (i.e., both hosted and invited) at baseline. Taken together, results provide support for continuing to teach autistic youth social skills via telehealth delivery.

In the telehealth modality, didactic lessons, role plays, behavioral rehearsals, and weekly assignments were translated to remote instruction and continued to be effective methods for improving youth social skills outcomes. In addition to providing support for telehealth interventions, results also highlight the continued need for evidence-based interventions for youth on the autism spectrum and parent-assisted interventions to promote generalization of skills. Core elements of the PEERS® program are parent-supported enrollment in social activities and regularly organized get-togethers with peers; the telehealth modality also translated social coaching to remote instruction and provided parents with strategies on how to support their adolescent’s social skills for in-person and online interactions. Regardless of treatment delivery, parents continue to be important for providing youth with opportunities to participate in social activities and generalizing skills outside of the treatment setting.

No significant differences were found between telehealth and in-person groups on most outcome measures except parent report of get-togethers, in that parents reported a greater increase in get-togethers following the telehealth format than following in-person instruction. A potential confound in assessment of social engagement with the QSQ is the addition of in-group get-togethers to the weekly assignments in our telehealth program to promote practice of online social skills and reduce social isolation due to COVID-19. Because of this adaptation, number of get-togethers reported may have been artificially inflated, as some participants may have included in-group get-togethers in the self- and parent-reports of number of get-togethers in the previous month. Despite this concern, it is still encouraging that participants continued to have get-togethers with peers unaffiliated with the program during a period of social distancing and remote learning. As a result, the UCLA PEERS program has since revised the QSQ to allow respondents report in- and out-group get-togethers separately; this will allow for the comparison of in-group and out-of-group get-togethers in future analyses of social engagement.

## Limitations and Future Directions

Although results from the present study are promising and merit further research, a few limitations should be considered when interpreting findings. Most importantly, families in the telehealth modality were recruited for participation in the virtual format. Ability to participate for the entirety of the program via telehealth may have impacted which families ultimately decided to enroll. Future studies may randomly assign families to either the in-person or telehealth format to assess efficacy and compare outcomes. Although there are many benefits to offering clinical services over telehealth (e.g., flexibility of scheduling, expanding reach into rural and remote areas), the telehealth modality also required access to reliable internet and technology (e.g., phone, tablet, laptop, or computer) for both the adolescent and parent. Availability of adequate internet and technology is a limiting factor for many families and can result in service disparities that must be addressed as healthcare providers continue to conduct services via telehealth. Secondly, the current study utilized outcome measures from participants who completed both baseline and post-intervention measures to determine efficacy of telehealth delivery; thus, data from participants who dropped from the telehealth program or who did not complete measures at both time points were not examined. Comparison of data from individuals who completed and did not complete the telehealth program may elucidate factors (e.g., scores on baseline measures, severity of social communication challenges, age) that impact successful completion of the telehealth modality, and thus, warrants further examination. Additional incentives, such as gift cards or prizes, or procedural adaptations may also be needed to ensure timely completion of outcome measures for telehealth participants.

Additional limitations must be considered. The current study included adolescents with historical ASD diagnoses and/or clinically elevated scores on the SRS-2. Future studies may include comprehensive diagnostic assessment utilizing standardized measures to confirm diagnoses and level of support needed. Supplementary measures of cognitive, adaptive, and psychological functioning, as well as receptive and expressive language ability, may also serve to better characterize the sample and assist in identification of moderator variables contributing to relative benefits of in-person or telehealth modalities. Additionally, given that the adolescents and parents are active participants in the intervention, their reports on outcome measures may be susceptible to bias. Researchers have found PEERS® may improve parent and family outcomes [e.g., parental stress, parenting self-efficacy, family chaos; (Corona et al., 2019; Karst et al., 2015), which may also influence respondent reports on treatment outcome measures. To address this, future research may collect third party (e.g., teacher report) and direct observational measures to objectively assess treatment outcomes and skills mastery in the youth’s natural settings. Such measures would be particularly beneficial if the respondent was blind to the adolescent’s participation in the intervention. Lastly, to further assess the efficacy of telehealth delivery, long-term follow up research is warranted to examine if outcomes similarly maintain over time as seen following the in-person program. Specifically, previous research on PEERS® for Adolescents has demonstrated durability of treatment gains, with outcomes maintaining 1–5 years after the conclusion of the intervention (Mandelberg et al., 2014). Follow-up assessments of adolescents participating in the telehealth program are needed to determine if improvements in social functioning also maintain following telehealth instruction.

## Conclusions

PEERS® for Adolescents via telehealth delivery demonstrated efficacy in improving social skills for autistic youth. Most encouragingly, outcomes from telehealth delivery were relatively equivalent to in-person treatment, suggesting that adolescents on the autism spectrum were able to learn important friendship skills in a remote learning context. Despite a challenging and uncertain social climate, adolescents were able to learn and exhibit significant gains in social skills knowledge, social responsiveness, overall social skills and problem behaviors, and social engagement. The present study provides support for ongoing implementation of telehealth delivery to expand accessibility of evidence-based interventions and to produce significant and meaningful social change in the lives of autistic youth and their families.

## Data Availability

Coded data for this study are available upon request from the UCLA PEERS Clinic.

## Footnotes

1 In accordance with autistic individual’s preference, identity-first language (i.e., “autistic”) and “on the autism spectrum” are used to describe the adolescent participants of the study throughout the manuscript (Bury et al., 2020)
